# Screening, Cloning and Expression of Active Streptokinase from an Iranian Isolate of *S.equisimilis* Group C in *E. coli*

**Published:** 2013-04

**Authors:** Malihe Keramati, Farzin Roohvand, Mohammad Mehdi Aslani, Shohreh Khatami, MohammadReza Aghasadeghi, Mehdi Sadat, Arash Memarnejadian, Fatemeh Motevalli

**Affiliations:** 1 Microbiology Department, Pasteur Institute of Iran, Tehran, Iran; 2 Virology Department, Pasteur Institute of Iran, Tehran, Iran; 3 Biochemistry Department, Pasteur Institute of Iran, Tehran, Iran; 4 Hepatitis and AIDS Department, Pasteur Institute of Iran, Tehran, Iran

**Keywords:** Gene expression, Recombinant streptokinase, Streptococcus

## Abstract

***Introduction: ***Streptokinase (SK) is a fibrinolytic protein secreted by β-hemolytic streptococci (βHS) groups A, C and G. Due to its importance as a thrombolytic drug, national screening programs in different countries for isolation of βHS and especially SK-producing group C (GCS) strains have been conducted. Herein, we provide data of the first screening study on βHS isolates in Iran for the aim of recombinant SK (rSK) production from a local strain.

***Materials and methods:*** 252 streptococcal samples were collected and characterized using microbial/biochemical assays. The GCS strains were serologically confirmed. Activity of GCS supernatant cultures was determined by caseinolytic assay in comparison with the standard strain GCS9542. The SK gene of the highest producer strain was selected for production of rSK in *E.coli* system. The rSKs activities were determined using chromogenic assay.

***Results: ***βHS were detected in 75 of the collected specimens (29.4%) including groups A (25.8%), C (3.6%) and G (0.4%). Analyses by SDS-PAGE and Western blotting indicated the proper expression of 47 kDa rSK proteins in *E. coli *for SK genes which were cloned from both the selected (GCS-87) and standard (GCS-9542) strains with the yields of 0.53 and 0.59 mg/ml (of the purified protein), respectively. The calculated activity for rSK 87 was around 90% of rSK9542 activity (0.18x105 IU/mg v/s 0.21x105 IU/mg).

***Conclusion:*** Results of the present study for the first time provided the possibility of producing rSK from a local and native source with comparable yields and activities similar to the standard strain.

## Introduction

Pathologic blood clots (in the form of thrombus) can result in vascular blockage which can induce serious consequences including death (1). In a healthy haemostatic system, formation of blood clots is suppressed through conversion of zymogen plasminogen (Plg) to plasmin (the serine protease that degrades fibrin) (2). However, in pathological conditions, clinical intervention through application of plasminogen activators (also known as “thrombolytic or fibrionolytic agents”) to relieve the vein from thrombosis is required. Currently, routine thrombolytic agents in clinical applications are recombinant human tissue plasminogen activator (tPA), urokinase (UK) and streptokinase (SK) (3). SK unlike UK and tPA activates Plg indirectly by complex formation and in a fibrin non-specific manner (4). SK exhibits significantly higher *in vivo *half-life compared to UK and tPA, but it’s fibrin non-specific mode of action and bacterial origin may increase the risk and side effects of thrombolytic therapies compared to the other two agents (2). Despite these short comings, SK gained a worldwide acceptance in developing countries due to its half-life, cost-effectiveness and shorter period of therapy (3). Moreover, a number of large-scale clinical trials, which have been conducted to compare the clinical efficacy of SK and tPA could not indicate a clear preference for either drug (5-6). Although most of the group A, C and G β-hemolytic streptococci (GAS, GCS and GGS respectively) produce and secrete SK , GCS which are neither erythrogenic toxin generators nor very fastidious in growth requirements, are the preferred bacteria for SK production (7). Historically, GCS strain *S. equisimilis *H46A (ATCC 12449) was the first streptococci to be introduced as a high-yield SK secreting bacteria (for production aims) by Christensen *et al* in 1945. Subsequently, Estrada *et al* introduced another *S. equisimilis* group C (ATCC 9542) as a SK production strain in 1992 (7). These two strains were used for SK production and served extensively as the principal source of SK gene for heterologous expression of the recombinant SK (rSK) in other hosts like *E. coli* and yeast (3).

**Table 1:** Characteristics of the bacterial samples identified in this study

Streptococci group	Throat culture	Genital tract	Urine culture	Skin	Blood culture	CSF	N.D	Total
GAS	60	-	4	1	-	-	-	65
GCS	7	2	-	-	-	-	-	9
GGS	1	-	-	-	-	-	-	1
GBS	51	20	27	3	1	3	4	109
GDS	19	-	8	3	4	-	2	36
Staphylococcus	8	-	-	-	-	-	3	11
Other streptococci	16	-	3	1	-	-	2	23
Total	162	21	42	7	3	3	11	252

N.D : not determined

Due to the clinical importance and increasing potential of SK application, a great deal of effort has been directed towards improvement of quality and quantity of SK production. Most of these studies were focused on either optimization of production conditions for *S. equisimilis* H46A and 9542 , or strain development using mutant strains or protein engineering using recombinant DNA technology (8-9). Sequencing studies on the SK genes and proteins from different isolates indicated that they are heterogeneous genes (10). In fact, the sequence identity of mature SK proteins with the same number of amino acids (414 residues), ranges from 80% to 98% (11). SK heterogeneity may reflect functional diversity of the gene products in pathogenesis, antigenic variation, solubility (12), Plg activation (13) and fibrinolytic activity (14). This implies that, alternatively, it may be possible to isolate SK protein(s) with better fibrinolytic characteristics that have clinical benefits by screening among different streptococci. In this context, a number of national screening programs to isolate SK producing β-hemolytic streptococci (βHS) from local and regional samples have been conducted in different countries (15-17). However, to our best of knowledge, there is no prior report on screening and characterization of SK producing strains or recombinant expression of SK from Iranian isolated streptococci strains.

In the present study, an attempt was made for isolation and characterization of βHS among Iranian clinical isolates to screen for the best SK producing GCS strains and to clone and express the corresponding SK gene in *E. coli*, for the final aim of SK production from local and native sources.

## Materials and Methods

*Bacterial strains and culturing conditions*

Two hundred and fifty two samples (initially assumed as streptococcal samples) were collected from patients with various non-invasive streptococcal diseases from different regions of Iran, during 2006 and 2010 (Table 1). *S. pyogenes* (GAS) ATCC 10403, *S.dysgalactiea spp.equisimilis* ATCC 9542 (GCS-9542) and *S.dysgalactiea spp.equisimilis *(GGS) CIP 55.120 (Pasteur institute of Paris) were used as reference strains for presumptive microbial, biochemical and serological tests (Table 2). All streptococcal isolates were cultured on 5% sheep blood agar and Todd-Hewitt Broth (THB) (Difco, USA) media. The plates were incubated at 37°C overnight. Colonies surrounded by alpha or beta-haemolysis were selected for more detailed characterization tests (Table 2). Presumptive standard identification tests including catalase test, susceptibility to a 0.04 U bacitracin disk and SXT disk (Sulfametoxazole 23.75µg –trimethoprim 1.25µg), CAMP test (Christie, Atkins, Munch-Petersen), PYR (Pyrolydonyl arylamidase) test, esculine hydrolysis, 6.5% NaCl tolerance and Voges- Proskauer (VP) tests were performed according to the standard protocols for determination of streptococci group A, C and G . Lancefield serotyping was performed by latex agglutination kit (Mast, UK). GCS Subspecies were further characterized by standard biochemical tests using ribose, sorbitol, lactose and trehalose fermentation (Table 2) (18). DH5α and M15 *E*. *coli *cells were cultured in Luria–Bertani (LB) medium. The pQE30 plasmid and M15 *E*. *coli *cells (Qiagen, USA) were used for cloning and expression of the SK gene and DH5α *E*. *coli *cells was used for propagation of plasmids. Kanamycin (50 µg/ml) was externally added to M15 *E*. *coli *culture.

**Table 2.** Microbial and biochemical analyses undertaken for the identification of beta hemolytic streptococci in our study (adapted from ref. 18)

	1	2	3	4	5	6	7	8	9
Haemolysis	β	β	β	β /α	β	β	β	β/ α	β /α
Growth in 6.5% NaCl	-	a	-	-	-	-	a	+	a
Growth in Bile-Aesculin	-	-	-	-	-	-	-	+	-
Voges-proskauer test	-	+	-	-	-	-	-	-	+
Pyrrolydonylarylamidase	+	-	-	-	-	-	-	+	-
Sensitive to bacitracin	+	-	v	v	-	-	-	-	-
H_2_O_2_ production	-	-	-	-	-	-	-	-	-
CAMP	-	+	-	-	-	-	-	-	+
Fermentation of Ribose	-	+	-	+	v	+	+	+	-
Sorbitol	-	-	-	v	+	-	-	-	-
Lactose	+	v	-	+	+	v	v	+	v
Trehalose	+	+	-	+	-	+	+	v	+
Lancefield antigen	A	B	C	C	C	C	G	D	F/C/A/G

1:*S. pyogenes*, 2:*S. agalactiae*, 3:*S. equi*, 4:*S. dysgalactiae,* 5:*S. zooepidemicus*, 6:* S. equisimilis*, 7:*S. spp.*group G (large colony variety), 8: *S. spp.*group D, 9: *S. anginosus*.

(α) Green zone around colonies on Blood Agar, (β) Clear, colourless zone around colonies on Blood Agar, (a) some strain will grow in 4% NaCl broth, (v) variable, (+) positive result, (-) negative result

**Figure 1.**
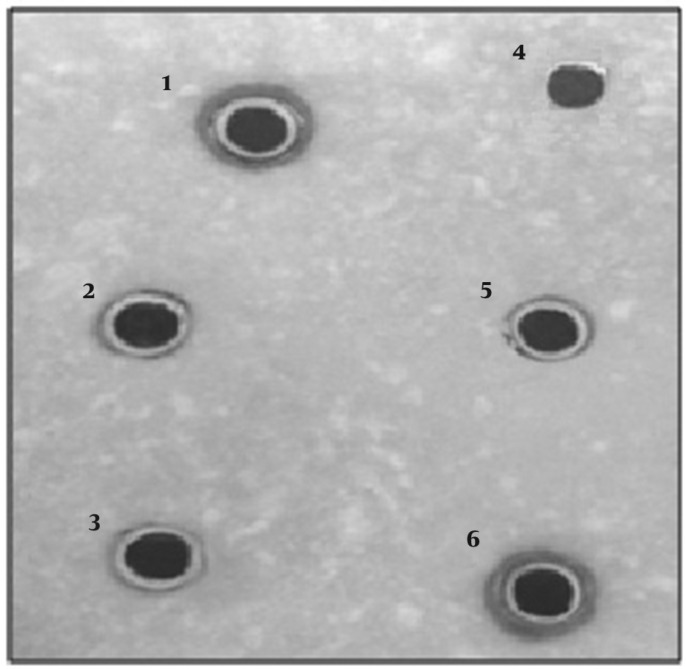
Semi-quantitative analysis of streptokinase activity by radial caseinolysis assay: Supernatant of GCS was used to fill the wells and THB was used as a negative control (well 4). GCS 9542 (well 1), GCS-S87 (well 6), GCS-S04, GCS- K17and GCS-K34 supernatants (well 2, 3 and 5) respectively

*Caseinolytic assay for streptokinase activity of bacterial culture*

Activity of streptococcal supernatant cultures was determined by caseinolytic assay(19) . Briefly, 50µl of overnight culture of stains in THB was added to 5ml of fresh THB and incubated at 37°C at 150 rpm. The culture supernatants were collected by centrifugation at mid-log phase (OD_600 _of 0.7-0.8) and were filtered using 0.22 µm PVDF filter (Whatman, Germany). The cell-free fluid was used to fill the pre-made wells in medium containing: 5 % skim milk and 1% agarose in sterile culturing plates. The same volume of human plasminogen (1 mg/ml) in a buffer containing: 150 mM NaCl and 50 mM Tris-HCl pH 7.4 was simultaneously added to the corresponding wells. Supernatant of* S.equisimilis* 9542 and THB were included as positive and negative controls in the corresponding wells, respectively. Plates were incubated overnight at 37°C. The clear area around the wells represented the level of SK activity of the corresponding strain. 

*Isolation of the streptokinase genes and plasmid construction*

**Figure 2:**
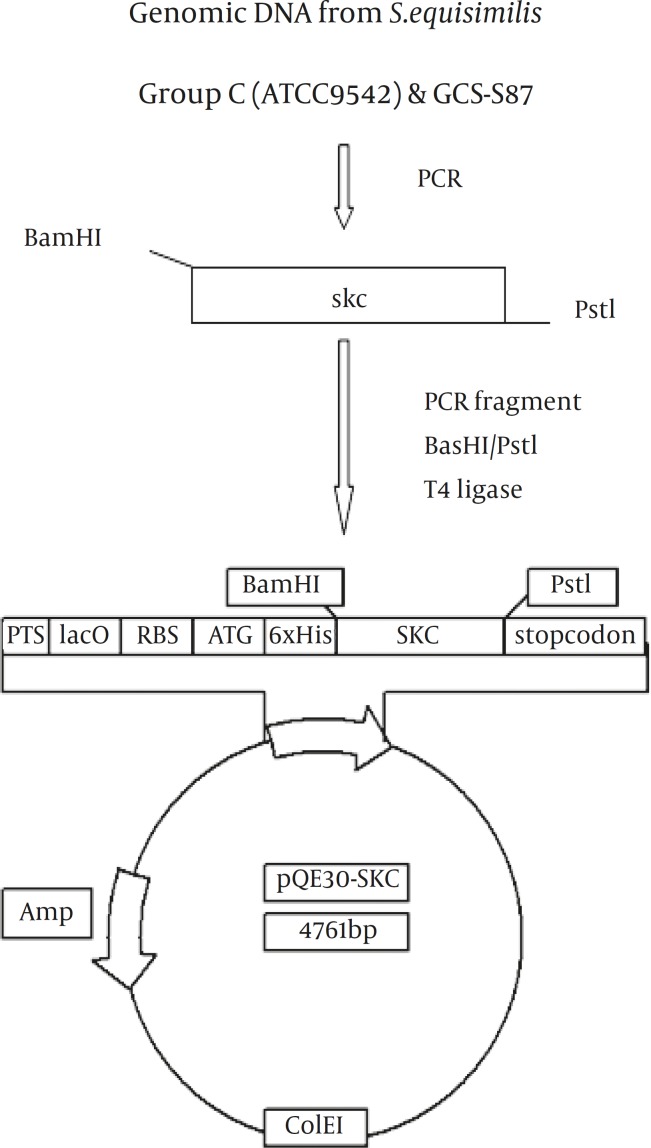
Construction of streptokinase expression plasmid pQE-SK

Genomic DNA of *S.equisimilis* (GCS-9542) and the selected GCS strain that showed the highest level of SK activity (GCS-S87) in caseinolytic assay (Figure1) was isolated by DNA extraction kit (AxyGene, USA) and used as a template for PCR-mediated isolation of SK genes. The coding region of SK gene (lacking the signal peptide sequence) was amplified by PCR using primers with inserted restriction sites for direct cloning into pQE30 vector (forward primer; *BamH*I-SKf: 5-TGGATCCATTGCTGGACCTGAGTGG CTG-3; reverse primer;* Pst*I-SKr: 5-CGCCGCAGTTATTTGTCGTTAGGGTTATC, the sequences corresponding to restriction sites are underlined). The resulting amplified fragments were digested with *BamH*I and *Pst*I and cloned into the same sites of pQE30 expression vector in tandem with the fused N-terminally 6XHis-tag and downstream of T5 promoter (Figure 2). Proper expression constructs were confirmed by restriction enzyme analysis and bidirectional sequencing. All cloning steps were performed according to standard procedures (20).

*Protein expression in E. coli*

*E. coli* M15 cells, which carry multiple copies of pREP4 plasmid that tightly regulate recombinant protein expression (21) were used as an expression host for pQE30 plasmids according to the manufacturer’s protocol (Qiagen,USA). Briefly, after transformation of bacterial cells with the recombinant plasmids pSK9542 and pSK87 using the standard CaCl_2_ method (20), expression of the target fusion protein was induced at OD_600_ of 0.5–0.6 by isopropyl-β-D-thio-galactoside (IPTG) to a final concentration of 0.5 mM. Cells were harvested by centrifugation after 6 hours of incubation at 37°C and stored at -20°C for purification steps.

The PCR amplified fragments corresponding to SK genes were digested with BamHI and PstI and ligated with the vector pQE30, previously digested with the same enzymes. PT5, LacO, RBS and ATG denote to T5 promoter, lactose operator, ribosome binding site and translation initiation code (ATG), respectively

*Purification and refolding of expressed SK*

The His6-SK fusion proteins were purified by nickel affinity chromatography under denaturing conditions according to the manufacturer’s protocol (Qiagen,USA). The pellet was dissolved in denaturing binding buffer (8 M Urea, 100 mM NaH_2_PO_4_, 100 mM Tris-Cl pH 8.0) and supernatant of the solubilized suspension after centrifugation was loaded onto Ni–NTA agarose resin. After washing steps, the fusion proteins were eluted with the elution buffer (8 M Urea, 100 mM NaH_2_PO_4_, 100 mM Tris-Cl t pH 4.5). The eluted proteins were refolded via dialysis in refolding buffer (20mM Tris-HCl pH7.4 and 10% Glycerol). Polyethylene Glycol (PEG) 20000 was used for protein concentration according to standard procedures (22). The protein concentration was determined by standard Bradford assay and optical density at 280nm (OD_280_).

*SDS-PAGE and Western blot analyses of rSK*

SDS–polyacrylamide gel electrophoresis was performed for protein expression assay. For western blotting, proteins were transferred to nitrocellulose membrane and the membrane was blocked by 5% BSA. Mouse anti-penta His monoclonal antibody (Qiagen, USA) was used as the primary antibody and goat anti-mouse IgG conjugated to HRP (Horse Radish peroxidase) (Qiagen,USA) as the secondary (tracking) antibody .The bound antibodies were detected using 3, 3- Diaminobenzidine (DAB) (Qiagen, USA). 

*Chromogenic assay of purified rSK activity*

SK activity was determined by chromogenic substrate as previously described (23). Purified rSK (5nM) was added to a microtiter plate containing 0.2 mM of chromogenic substrate S-2251 (H-D-valyl-L-leucyl-L-lysine-p-nitroanilide dihydrochloride; Sigma, USA) and 200 nM of human plasminogen (Sigma,USA) at 37 °C in a total volume of 100 µl of assay buffer (50 mM Tris-HCl, 150 mM NaCl, pH 7.4). Hydrolysis of S-2251 was measured at 405 nm every 5 min for 60 min in a microplate reader (BioHIT, UK). The protein activity was calculated using standard activity curve of streptase^®^ (CSL, Behring, Germany).

## Results

*Screening of β-haemolytic streptococci (βHS) and isolation of SK producing GCS*

A total of 252 samples (Table 1) were examined by microbiological and biochemical assays (Table2). βHS including group A, C and G were found in 75 out of 250 streptococci specimens (29.4%). Group A was the dominant Lancefield serogroup found in 65 out of 250 streptococci specimens (25.8%) followed by GCS (9 out of 250; 3.6%) and GGS (1 out of 250; 0.4%), respectively. Throat culture was the common source of GAS (60 out of 250 isolates), followed by urine culture (4 out of 250 isolates) and soft tissue (1 out of 250 isolates). GCS were less common and were totally isolated from respiratory tract (7 out of 250 isolates) and genital tract (2 out of 250 isolates). More detailed fermentation analyses on isolated GCS (Table 2) could determine eight *S. dysgalactiae* subsp. *equisimilis* strains and one strain of *S.dysgalactiae* subsp. *dysgalactiae *(Table 3; sample codes are based on in house coding). Most of GCS isolates showed low to moderate SK activity in caseinolysis assay, except for GCS-S87 that showed predominant SK activity compared to the reference strain (GCS-9542) and thus was selected for further cloning studies (Figure 1). 

**Table 3.** Distribution of identified GCS subspecies in different samples

No.	Sample No.	culture Source	Disease	Sub species of GCS
1	S-04	Human throat	Streptococcal pharyngitis	*S.equisimilis*
2	S-05	Human throat	Streptococcal pharyngitis	*S.equisimilis*
3	S-08	Human vagina	Puerperal fever	*S.equisimilis*
4	S-87	Human throat	Streptococcal pharyngitis	*S.equisimilis*
5	S-91	Human throat	Acute tonsillitis	*S.dysgalactiae*
6	S-131	Human vagina	Puerperal fever	*S.equisimilis*
7	K-17	Human throat	Acute tonsillitis	*S.equisimilis*
8	K-19	Human throat	Acute tonsillitis	*S.equisimilis*
9	K-34	Human throat	Acute tonsillitis	*S.equisimilis*

**Figure 3:**
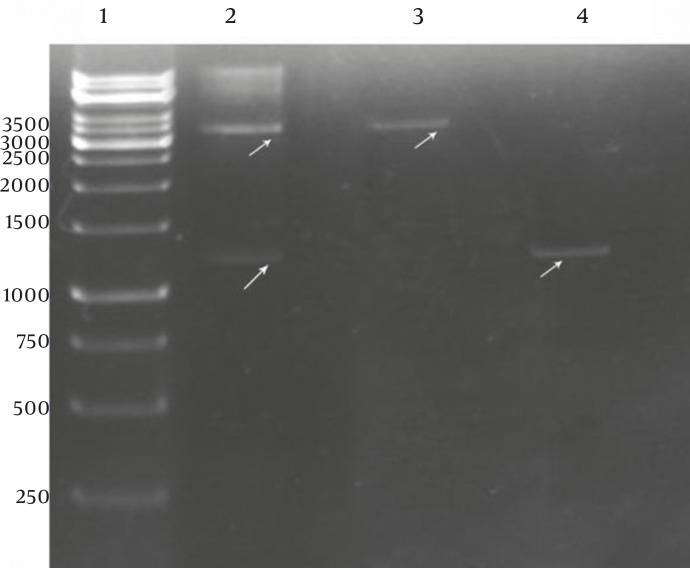
Restriction Enzyme Analysis of Recombinant pQE-S87

*Cloning, expression, purification and refolding of the rSK*

Using the SKf and SKr primers and genomic DNA of *S.equisimilis* GCS-9542 and *S.equisimilis* GCS-S87 as template, PCR reactions resulted in a single band of the expected length (1242bp) of SK gene for both strains (Figure 3). Cloning steps for insertion of SK gene in pQE30 vector is illustrated (Figure 2). Restriction enzyme analyses of the recombinant vector harboring SK gene (pSK87) (Figure 3) and nucleotide sequence analyses (not shown) confirmed the accuracy of cloning procedures. 

**Figure 4:**
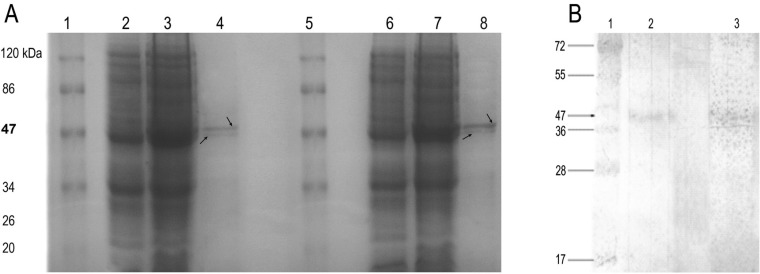
Analysis of recombinant proteins by SDS-PAGE and western blot

Expression of pSKC plasmids produced rSK with a predicted molecular mass of about 47 kDa, harboring a 6XHis domain which was appended to the N- terminus of the native molecule. Crystal structure of streptokinase shows that the N-terminal of the enzyme is unfolded (24), thus, addition of polyhistidine at the NH_2_-terminus of the enzyme was expected to have little or no effect on the catalytic activity. Analysis of protein profile of un-induced and induced cell lysates on SDS-PAGE proved expression of SK by both strains (Figure 4A). Analysis of the purified rSK further indicated two major bands on the gel (Figure4A). The upper band which was the most prominent protein corresponded to the full length SK (47 kDa) and the lower band (around 44 kDa) might be related to the digested form of SK as previously suggested (25). Accordingly, the eluted (purified) recombinant proteins were identified by the presence of the same two bands in western blot analysis (Figure 4B). Evaluation of the expression efficiencies for the rSK proteins by concentration measurements at OD_280_ and Bradford assay indicated yields of 0.53 mg/ml (rSK9542) and 0.59 mg/ml (rSKS87) for the purified proteins.

*Biological activity assay of streptokinase by chromogenic method*

The chromogenic assay in the absence of fibrin is known as an approved an internationally standard assay for streptokinase activity (Third International Standard for streptokinase; National Institute of Biological Standard and Controls, NIBSC, 2004UK) (26). Employing this method and chromogenic substrate S-2251, a standard curve based on definite activity of Streptase^®^ was plotted (Data not shown). Subsequently, the biological activity (which represents the activity of SK in International Units per ml of total volume; IU/ml) and specific activity (which refers to the activity of rSK per mg of total protein; IU/mg) were calculated based on the plotted standard curve. The calculated values for the biological activities were 11200 IU/ml (rSK9542) and 10720 IU/ml (rSKS87) and for specific activities were 0.21x10^5^ IU/mg (rSK9542) and 0.18x10^5^ IU/mg (rSKS87). 

Panel A: SDS–PAGE (12%) analysis of protein expression and purification; Lanes 1and 5: protein marker; lanes 2 and 3: total protein extracts from E. coli M15/pSK87 and E. coli M15/pSK9542 (lanes 6 and 7). Lanes 3 and 7 corresponds to IPTG induced and lanes 2 and 6 correspond to un-induced bacterial cells. Lane 4 and 8 represent purified proteins of rSK87 and rSK9542 respectively. The position of the 47 and 44 kDa bands are indicated by arrows. Panel B: Western blot analyses by anti-His monoclonal antibody; lane 1: MW protein marker, lane 2: rSK9542 and lane 3: rSKS87

## References

[1] Anmol Kumar AK, KK Pulicherla KKP, K. Seetha Ram KSR, KRS Sambasiva Rao KRSSR (2010). Evolutionary trend of thrombolytics. Int J Bio Sci Bio Technol.

[2] Banerjee A, Chisti Y, Banerjee UC (2004). Streptokinase--a clinically useful thrombolytic agent. Biotechnol Adv.

[3] Kunamneni A, Abdelghani TTA, Ellaiah P (2007). Streptokinase—the drug of choice for thrombolytic therapy. J Thromb Thrombolysis..

[4] Baruah DB, Dash RN, Chaudhari M, Kadam S (2006). Plasminogen activators: A comparison. Vasc Pharmacol.

[5] Della G, Miocardico SNI (1990). GISSI-2: a factorial randomised trial of alteplase versus streptokinase and heparin versus no heparin among 12,490 patients with acute myocardial infarction. Lancet.

[6] Ridker PM, O’Donnell C, Marder VJ, Hennekens CH (1993). Large-scale trials of thrombolytic therapy for acute myocardial infarction: GISSI-2, ISIS-3, and GUSTO-1. Ann Int Med.

[7] Sikri N, Bardia A (2007). A history of streptokinase use in acute myocardial infarction. Texas Heart Institute J.

[8] Abdelghani TTA, Kunamneni A, Ellaiah P (2005). Isolation and mutagenesis of streptokinase producing bacteria. Am J Immunol.

[9] Arabi R, Roohvand F, Noruzian D, sardari S, Aghasadeghi MR, Khanamad H (2011). A comparative study on the activity and antigenicity of truncated and full-length forms of streptokinase. Polish J Microbiol.

[10] Malke H (1993). Polymorphism of the streptokinase gene: implications for the pathogenesis of post-streptococcal glomerulonephritis. Zentralblatt für Bakteriologie. Int J Med Microbiol.

[11] Malke H, Steiner K, Gase K, Mechold U, Ellinger T (1995). The streptokinase gene: allelic variation, genomic environment and expression control. Dev Biol Stand.

[12] Pupo E, Baghbaderani BA, Lugo V, Fernández J, Páez R, Torréns I (1999). Two streptokinase genes are expressed with different solubility in Escherichia coli W3110. Biotechnol lett.

[13] Keramati M, Roohvand F, Eslaminejad Z, Nikbin VS, Aslani MM (2012). PCR/RFLP-based allelic variants of streptokinase and their plasminogen activation potencies. FEMS Microbiol Lett.

[14] Tewodros W, Norgren M, Kronvall G (1995). Streptokinase activity among group A streptococci in relation to streptokinase genotype, plasminogen binding, and disease manifestations. Microbial Pathogenesis.

[15] Al Sohaimy S, Aleem E, Hafez EE, Esmail SS, El-Saadani M, Moneim NA (2011). Expression of recombinant Streptokinase from local Egyptian Streptococcus sp. SalMarEg. Afr J Biotechnol.

[16] Freeda F (2010). Production and partial purification of streptokinase by streptococcus sp. Int J Students Project (IJSP)-Biotechnol.

[17] Doss HM, Manohar M, Singh NA, Mohanasrinivasan V, Devi CS (2011). Studies on isolation, screening and strain improvement of streptokinase producing -hemolytic streptococci. World J Sci Technol.

[18] Garrity GM, Brenner DJ, Krieg NR (2005). Bergey’s manual of systematic bacteriology.

[19] Kim DM, Lee SJ, Kim IC, Kim ST, Byun SM (2000). Asp41-His48 region of streptokinase is important in binding to a substrate plasminogen. Thrombosis Res.

[20] Sambrook J, Russell DW (2006). The condensed protocols from molecular cloning: a laboratory manual.

[21] Crowe J, Henco K (1992). The QIAexpressionist. DIAGEN GmbH.

[22] Ingham KC (1990). Precipitation of proteins with polyethylene glycol. Methods Enzymol.

[23] Wohl RC, Summaria L, Robbins K (1980). Kinetics of activation of human plasminogen by different activator species at pH 7.4 and 37 degrees C. J Biologic Chem.

[24] Wu XC, Ye R, Duan Y, Wong SL (1998). Engineering of plasmin-resistant forms of streptokinase and their production in Bacillus subtilis: streptokinase with longer functional half-life. App Environ Microbiol.

[25] Pimienta E, Ayala JC, Rodríguez C, Ramos A, Van Mellaert L, Vallín C (2007). Recombinant production of Streptococcus equisimilis streptokinase by Streptomyces lividans. Microb Cell Fact.

[26] Sands D, Whitton C, Longstaff C (2004). International collaborative study to establish the 3rd International Standard for Streptokinase. J Thromb Haemost.

[27] Winn WC, Koneman EW (2006). Koneman’s color atlas and textbook of diagnostic microbiology.

[28] Hughes JM, Wilson ME, Brandt CM, Spellerberg B (2009). Human infections due to Streptococcus dysgalactiae subspecies equisimilis. Clin Infect Dis.

[29] Barnham M, Kerby J, Chandler R, Millar M (1989). Group C streptococci in human infection: a study of 308 isolates with clinical correlations. Epidemiol Infect.

[30] Davies MR, McMillan DJ, Sriprakash KS, Chhatwal GS (2006). Distribution of group A streptococcal virulence genes in group C and G streptococci. Int Cong Series.

[31] Steiner K, Malke H (2002). Dual control of streptokinase and streptolysin S production by the covRS and fasCAX two-component regulators in Streptococcus dysgalactiae subsp. equisimilis. InfectImmun..

[32] Kim MR, Choeng YH, Chi WJ, Kang DK, Hong SK (2010). Heterologous production of streptokinase in secretory form in streptomyces lividans and in nonsecretory form in escherichia coli. J Microbiol Biotechnol.

[33] Balagurunathan B, Ramchandra NS, Jayaraman G (2008). Enhancement of stability of recombinant streptokinase by intracellular expression and single step purification by hydrophobic interaction chromatography. Biochem Engineering Journal..

[34] Longstaff C, Thelwell C, Whitton C (2005). The poor quality of streptokinase products in use in developing countries. J Thromb Haemost.

